# Spontaneous Conception in Couples Who Need Assisted Reproduction Technology Treatment—A Narrative Review

**DOI:** 10.3390/medicina62071230

**Published:** 2026-06-25

**Authors:** Izhar Ben Shlomo, Dikla Kamisa, Vardi Benesh Raviv

**Affiliations:** 1 The Program for Emergency Medicine, Zefat Academic College, Safed 1320611, Israel; 2 The Azrieli Faculty of Medicine at Safed, Bar Ilan University, Ramat Gen 5290002, Israel; 3 Program of Nursing, Zefat Academic College, Safed 1320611, Israel

**Keywords:** spontaneous conception, assisted reproduction technology, infertility etiology

## Abstract

*Importance*: Most couples who turn to assisted reproductive technology (ART) treatment do so, usually, after giving up emotionally on the chances of conceiving naturally. Others undergo ovulation induction with intrauterine insemination (IUI) and turn to ART after the latter has failed. Spontaneous conception after having experienced the exhausting process of ART, whether it was successful or not, could be very surprising and confusing for many couples. *Objective*: Review all the scenarios within which an unexpected spontaneous conception can occur and the likelihood of its occurrence. These are four such scenarios: (1) after being referred to ART but before the actual initiation of ART; (2) between ART cycles; (3) after a successful ART pregnancy; (4) after giving up on treatment. We have found only a systematic review on #3, but not the other three. *Evidence Review*: We collected all PubMed citations for the terms “spontaneous conception” and ART or IVF. Thereafter, we realized that no AI tool can filter only the relevant literature. Hence, we exhausted all possible cross-references by manual search to ensure the completeness of the search. *Findings*: In each of the four scenarios, spontaneous conceptions occur. Before treatment, a critical element is the length of the waiting time, as is the gap between treatments when already treated, with the cost of treatment being a critical determinant. After the conclusion of treatment, whether successful or failed, the main determinants of the chance for spontaneous conception are age, length of infertility, and the leading etiology for infertility. Overall, the chances range from as little as 2% and up to 25%, with severe male factor and a woman’s age being the most notable for low rates. *Conclusion and Relevance*: Each couple entering ART treatment should be informed of the chances for spontaneous conception, whether as an aid to the decision to enter or the decision to leave after a failure, and on the more cheerful side, to be aware of the chances for unplanned pregnancy after a successful treatment.

## 1. Introduction

Most couples who turn to assisted reproductive technology (ART) treatment do so after giving up emotionally on the chances of conceiving naturally or being advised to resort immediately to it. Some other couples undergo the less-demanding ovulation induction with intrauterine insemination (IUI) and turn to ART after the latter has failed. Spontaneous conception after having experienced the exhausting process of ART, whether it was successful or not, could be very surprising and confusing for many couples. Moreover, some couples conceive spontaneously right after being referred to ART. For some couples, it may be a very blessed and welcomed event, whilst for others, it could be after they “came to terms with their fate” and closed the chapter of reproduction and family planning, and already put the pain of not having a child behind them. However, even among those who embrace the occasion, it can raise questions as to whether the undoubtedly demanding process they already went through was necessary, either emotionally or economically, or whether the clinical judgment of the doctors was correct. A significant contributing factor to this already complex reality is the accessibility of ART. In some countries, the process is very expensive, whereas in others it is state-sponsored or paid for, partially or fully. Yet, even when state-sponsored, in some countries, it may be regulated as to the frequency of treatments or the minimum length of infertility to allow treatment.

In short, there are four scenarios within which a spontaneous conception can occur in ART patients: (1) after being referred to ART but before the actual initiation of ART; (2) in between ART cycles; (3) after a successful ART pregnancy; (4) after giving up on treatment. There have been publications on these scenarios, but we have found no systematic summary of all these combined. We, hence, do not doubt that all clinicians need to have an authoritative summary of this matter, to be able to give their patients a sincere, full scope of their chances to conceive with or without ART.

Furthermore, not only does the need for informed consent whilst practicing in the legal era exist but also the medical decency towards couples with a desperate will warrant the inclusion of the chances of subsequent spontaneous pregnancy in counseling couples who are considering the use of ART. Added to that is counseling for the need of contraceptives, particularly when there are obstetric implications such as Cesarean section in previous delivery or the development of a new medical entity in the mother that can be affected by or affect a future pregnancy. There is also a need for practitioners to reflect on what is the true contribution of their involvement, specifically when treatment is protracted in time, during which spontaneous conceptions may occur. In this regard, it is interesting that, in 2011, Brandes et al. [1] reported that, from a cohort of 1391 infertile couples followed for 2 years during treatments ranging from ovulation induction to IVF, 1001 conceived (72% pregnancy rate), of which 45.6% were spontaneous conceptions.

## 2. Method

We have searched PubMed, which includes only peer-reviewed, dependable publications, for the terms “spontaneous conception” and IVF or ART. Specifically, in light of the eclectic nature of the reports, none of which was the result of prospective cohort data collection, we felt that only those publications filtered through the peer-review process of well-recognized journals should be included (included in PubMed). We then deleted all the vast literature that compares obstetrical outcomes between pregnancies resulting from ART and spontaneous pregnancies. To exhaust the possibility of missed publications, we manually reviewed the reference lists of all retrieved articles to find further primary sources of information. Altogether, we ended up with 32 full-text publications on this matter. We have not used algorithmic selection tools, as we realized that our human examination of reference lists was better, especially in identifying the reports that comply with the desired profile, providing numerical data. For each article, we read for data regarding each of the four scenarios wherein spontaneous conception may occur and allocated the relevant numbers where they belong. Thus, a given publication may be mentioned in the summary of more than one of the four scenarios. We summarized the content of the publications chronologically, out of awareness of the constantly changing arena of ART.

As early as 1983, Collins et al. [2], in a key publication in the NEJM, found in a cohort of 1145 infertile couples that 61% of all pregnancies reported in a time span of 5 years were treatment-independent, indicating the need for well-focused studies of all relevant subgroups within the general infertile cohort.

The results of our review are presented chronologically, as methods of ART underwent several major leaps during the last five decades, which should be considered upon such a review.

### 2.1. Conceptions After Being Referred to but Before Actual Treatment by ART

Haney et al. [3] in 1987 enrolled 245 couples in their IVF program during 1983–1986. Four spontaneous conceptions occurred after enrollment in an IVF trial. Matorras et al. [4] in 1996 reported a two-year follow-up of couples waiting for intrauterine insemination by donor sperm. Among 86 couples with oligozoospermia and 33 with severe asthenozoospermia, 7.6% spontaneous pregnancies were recorded, but none among 166 couples with azoospermic partners. Eijkemans et al. [5] in 2008, using data on 5962 couples in the waiting period before the start of IVF, found that the cumulative rate of treatment-free ongoing pregnancy was 9% in a full year’s time. The chances for a spontaneous conception were, in an escalating order, endometriosis, tubal factor, male factor, hormonal, immunological, and unexplained infertility, reaching 25% in the latter. van Dongen et al. [6] in 2010 reported that, out of 674 couples on an ART waiting list, 13% dropped out before starting their first IVF cycle. Out of 85 respondents to targeted questioning, 37% dropped out because of spontaneous pregnancy.

All told, the duration from referral to the start of ART treatment, whether due to state regulation or other constraints, such as cost, has a significant impact in this “window” of opportunity to conceive, as does the woman’s age and the underlying medical cause.

Limitations: There are countries where ART treatment is fully self-paid, and therefore, the decision to embark on such a treatment is economically heavy and may take time until the actual start. In other countries or specific centers, the process of recruitment of couples may be more protracted than in other countries or centers. In the latter, the source of funding is also a factor, as when state-paid, it might entail more stringent criteria. We have not found a detailed description in any publication of the time from referral to treatment.

### 2.2. Spontaneous Conceptions Between ART Cycles

Roh et al. [7] in 1987, in a very early study before the era of ICSI, compared a group of 274 women who underwent 492 IVF treatment cycles regarding the rate of treatment-dependent versus -independent conceptions. They found the former to be 15% and the latter 6.6%, with a mean observation interval of two years. Of all spontaneous conceptions, 83.3% occurred within 6 months of the last IVF trial. Harrison et al. [8] in 1995 reported on treating 662 couples by IVF in Ireland, which resulted in 187 IVF pregnancies. Of the whole group, 78 conceived spontaneously. Interestingly, among the IVF pregnancies, the miscarriage rate was 16.5%, while only 5% miscarried among the spontaneous conceptions.

Almagor et al. [9] in 2001 reported on the pregnancies that occurred in 85 couples who underwent 119 ICSI cycles for severe oligoasthenozoospermia over mid-1996 through end-1997. Following ICSI, there were 27 clinical pregnancies (22.7%) pregnancy/cycle with only 11% deliveries, whereas 7 spontaneous clinical pregnancies occurred in 5 couples (6.5%). No breakdown for whether the spontaneous conceptions occurred after cessation or in between cycles was given, but the short time lapse can be taken to indicate that these occurred during waiting periods. Of 888 couples eventually treated by Donckers et al. [10], who reported in 2011, 220 (24.7%) conceived spontaneously between ART treatment cycles.

Patil [11] reviewed in 2022 the files of 329 patients who could not receive ART treatment because of the COVID-19 pandemic for 11 months during 2020. There were 31 (9%) spontaneous pregnancies among them. The scope of etiologies for infertility was materially different from that in the industrialized world, but none deviated much from the general 9%. Whether this represents spontaneous conceptions in between active treatment cycles is questionable, yet it sheds some light on the question of whether the gap between cycles derives from specific, local availability issues that affect this phenomenon.

All in all, the rate of spontaneous conceptions that occur between ART cycles ranges from some 7% to as high as 25%. We found no breakdown as to the etiologies of the couples who conceived spontaneously.

Limitations: Centers for ART usually are less interested in what happens between cycles when it comes to reporting this issue. Thus, the number of these reports is limited, probably representing under-reporting of such conceptions.

### 2.3. Spontaneous Conceptions After Successful ART Pregnancy

Shimizu et al. [12] documented, in 1999, spontaneous conceptions after the births of infants conceived by IVF in 299 deliveries, occurring from 1988 to 1994. The cumulative conception rate at 5 years after successful IVF was 18%, with most conceptions occurring within 2 years of delivery. They found that the most important factor in this occurrence was the woman’s age, followed by endometriosis, a mild male factor, or unexplained infertility. Hennelly et al. [13] surveyed, in 2000, 530 couples who had successful pregnancies after ART. They reported a total of 20.7% spontaneous conceptions within 8 years from the index pregnancy. Younger women and those with no sperm quality problems were more likely to conceive, even though a few of those who needed intracytoplasmic sperm injection (ICSI) also conceived. Women with unexplained infertility and endometriosis were also more likely to conceive after a successful ART pregnancy. Hennelly et al. [13] summarized in 2000 the response of 513 couples, out of 530 addressed, for subsequent spontaneous conception after successful IVF/ICSI treatment. The overall spontaneous conception rate was 20.7%. Younger women with shorter previous infertility were more likely to conceive, as were patients with unexplained infertility and endometriosis. They further reported that only 13 (2.5%) of the couples who needed ICSI conceived spontaneously afterwards.

Ludwig et al. [14] reported in 2008 on the chances of spontaneous conception among 1614 couples, of whom only 899 (55.7%) responded to a mail questionnaire. Of the respondents, 20% of those who wished to conceive succeeded in this, and of whom, for 74.5%, it occurred within 2 years of the index pregnancy. In a study by Pinborg et al. [15] in 2009, of the 817 women who provided questionnaire data on 5 years’ time lapse, 18.2% (149/817) delivered after spontaneous conception, two-thirds of whom were after a previous ART-dependent delivery.

Troude et al. [16] summarized in 2012, the cases of 2134 couples after departure from IVF, with 1320 that succeeded and 814 that did not. The birth rate after spontaneous conception following a previously successful IVF was 17% within 7–9 years. Young age and fewer IVF cycles were positive predictors of spontaneous conception. Out of 297 women who conceived their first pregnancy by ART, 236 responded to a questionnaire sent by Wynter et al. [17] in 2013 within two years after delivery. Of these, 33% reported a subsequent spontaneous conception. Those who conceived were, significantly, under the categories of unexplained infertility and shorter partner relationship duration. Lande et al. [18] in 2012 inquired as to what the rate of spontaneous live births after successful IVF treatment in a cost-free environment was and whether couples who achieved a spontaneous live birth were initially referred prematurely. They studied women aged <35 years with primary infertility who were referred for their first IVF treatment between 2001 and 2002 and followed up for 7 years for spontaneous live birth rate following successful ART. Of 109 patients who conceived by ART, 7 were excluded for not attempting further pregnancy. Of the 102 who desired further pregnancy, 22 (21%) conceived spontaneously and delivered a child. No number of conceptions that ended with miscarriage was reported. The referral to ART for those who later conceived spontaneously was not earlier than for those who did not conceive spontaneously.

Volgsten and Schmidt [19] reported in 2017 spontaneous conception and subsequent birth in 26.1% of women who had a live birth after successful ART. This report is very fragmented and does not allow for a full reconstruction of data according to our predefined windows of opportunity. No number of miscarriages was given in this report. El Mokhallalati et al. [20] reviewed, retrospectively, in 2019, the files of 2133 couples who conceived by ART treatment and found, within 5 years, that the live birth rate for spontaneous conception among women whose ART treatment was unsuccessful was 17%, compared to 15% in those whose ART treatment succeeded. No number of miscarriages was given in this report also. In both groups, young age, duration of infertility, and IVF compared to ICSI were positive predictors of spontaneous conception.

Alley et al. [21] in 2022 excluded from their analysis couples with tubal factor, azoospermia or primary ovarian insufficiency and found, among 2915 couples, 22% live births due to spontaneous conception. No number of miscarriages was given in this report. Most births (65%) were from patients after a previous successful ART. Successful ART, male factor, ovulatory dysfunction, or unexplained infertility were positively associated with spontaneous conception chances, while increasing maternal age and having a child before ART (probably in itself associated with advanced age) were negatively associated with it.

Thwaites et al. [22] performed a meta-analysis in 2023 of 11 studies, which included 5180 couples who succeeded on a previous ART treatment. Among these, the spontaneous delivery rate (underestimating the spontaneous conception rate) was 20% within 2–15 years of follow-up. No breakdown or analysis of the chances under certain diagnoses was provided. Moreover, the authors themselves rated the soundness of the data as insufficient.

Başbuğ et al. [23] compared, in 2024, 48 women who first conceived with ICSI and then achieved spontaneous pregnancy and found that gestational diabetes was higher in the spontaneous pregnancies, whereas amniotic fluid abnormality, gestational hypertension, and preterm delivery were higher in the ICSI group.

In summary, the chance to conceive spontaneously after successful ART ranges between 15% and 25%. The most important predictors of such a conception were the women’s age, the duration of previous infertility and the absence of absolute blockade to conception, such as tubal occlusion or azoospermia.

Limitations: The collection of data for all these reports leans on self-reporting of those who agreed to respond. This most obviously overlooks those who might have hard feelings towards the specific center, whether due to subsequent spontaneous conception (being interpreted as exposing the fraud of the diagnosis and the center) or due to other personal aspects of the interaction with the personnel involved. In addition, some probably have never received the contact letter, as people change addresses not infrequently.

### 2.4. Spontaneous Conceptions After Giving up on ART Treatment

In a very early era, when oocyte retrieval was done by laparoscopy, Ben-Rafael et al. [24] reported in 1981 that, of 149 patients who failed to conceive on IVF, 75 suffered from bilateral tubal blockage and none had conceived spontaneously. Of the remaining 74 women with at least one patent tube, 13 (17.5%) conceived spontaneously. Haney et al. [3] reported in 1987 on 245 couples enrolled in their IVF program during 1983–1986. The length of follow-up depended on the enrollment date and was therefore relatively short. Twelve spontaneous conceptions occurred after an unsuccessful IVF attempt. Osmanagaoglu et al. [25] reported in 1999 that, out of 149 couples who left after the failure of ICSI, spontaneous conception occurred in 23 couples (15.4%). The follow-up lasted up to 5 years. Of the 76 couples interviewed by Filetto & Makuch [26] in 2005 and who had not undergone any further treatment during the ensuing 3–8 years after failed IVF, eight women (10.5%) conceived spontaneously.

Osmanagaoglu et al. [27] reported in 2002 a 5-year follow-up in 200 couples who left IVF-ICSI treatment. They found 23 spontaneous pregnancies ending in delivery (11.5%). The mean time interval from last treatment to spontaneous pregnancy was 20.2 ± 13.7 months. Cahill et al. [28] examined in 2005 the likelihood of natural conception when a couple had ceased to try to conceive by ART between the years 1987 and 1991 and found among 116 couples (only 44% of those originally addressed) who responded that 18% conceived within a median of 7 months (range 1–84 months) from cessation of treatment. The likelihood of conception was affected by infertility diagnosis (from lower to higher tubal, endometriosis, sperm problem, unexplained, and other), the woman’s age, and duration of infertility, while primary infertility did not.

De La Rochebrochard et al. [29] reported in 2009 on 420 patients who concluded ART without success. Of these, only 216 were reached for information. Of these, 41 (19%) conceived spontaneously. The inferred follow-up period was about 5 years, but it was not specifically calculated. Brandes et al. [1] reported in 2011 on 104 couples diagnosed as unexplained infertility who left all fertility treatment, with 26 on doctor’s advice, of whom 4 (15.4%) conceived spontaneously, and 78 on their own initiative, of whom 33 (42.3%) conceived spontaneously. The time span for this data collection was not clearly disclosed, but since entry to the cohort began in 2002 and the publication was in 2011, this time span can be assumed to last a mean of 6–8 years.

Troude et al. [16] reported in 2012 sending questionnaires to 814 couples who departed from ART following failure and reported a 24% birth rate after spontaneous conception. Young age and fewer IVF cycles were positive predictors of spontaneous conception. Troude et al. [30] summarized in 2016 the files of 6507 couples who were treated in 8 French centers and found that, after 8 years from enlistment, 48% achieved pregnancy by treatment, whereas another 12% conceived spontaneously after giving up on treatment.

An internet-based questionnaire found among 403 eligible responders addressed in 2016 by Marcus et al. [31] that the overall cumulative live birth rate over a 6-year period following the cessation of treatment was 29%, with 82% of these occurring within 2 years. No number of miscarriages was given in this report. The factors associated with spontaneous conception were unexplained infertility, ovulatory dysfunction, and infertility of less than four years prior to IVF/ICSI. Volkstein & Schmidt [19] reported in 2017 on a relatively small group having 32.9% spontaneous conceptions with birth after failing ART treatments. This report is very fragmented and does not allow for a full reconstruction of data according to our predefined windows of opportunity.

Villani et al. [32], who called in 2021 to reschedule 431 couples to ART after the Italian lockdown for COVID-19, which lasted from 9 March to 4 May 2020, found that, within less than 2 months, 34 (7.9%) conceived spontaneously upon waiting. Age and shorter previous infertility were positive predictors, as well as increased sexual activity during the lockdown. Pacchiarotti et al. [33] found in 2020, during the same Italian lockdown, that 7 out of 50 couples (14%) conceived spontaneously after experiencing a mean of 2 years of infertility. Phone contact confirmed a significant increase in sexual activity among these couples.

In summary, the reasons for departing from ART that failed to provide a child are multiple, ranging from the physician’s recommendation to couples’ frustration, whether early or late. The subsequent conception rate ranges from some 5% up to 20%, conceivably due to variability in inclusion and the inconsistency of replies to questionnaires. Here, again, the age of the woman and the specific diagnosis of infertility are important. For many non-absolute causes of subfertility, spontaneous conception remains biologically possible and sometimes clinically relevant. However, in complete anatomical obstruction or gamete-absence conditions, the probability is expected to be very low or effectively negligible unless the underlying condition changes or is corrected.

Limitations: All that was relevant to the limitations in the window after a successful ART conception is relevant here, in addition to the understandable frustration involved in this window.

All four Scenarios are depicted in Table 1.

## 3. Implications for Counseling and Shared Decision-Making

All the above data should not be used merely as a disclosure statement. They may support shared decision-making at several points: before ART initiation, between cycles, after unsuccessful cycles, after an ART live birth, and when treatment is discontinued. In each setting, clinicians should explain that estimates are conditional on age, duration of infertility, diagnosis, time at risk, and whether the endpoint is conception, clinical pregnancy, ongoing pregnancy, delivery, or live birth. Specifically, people who enter under the title “unexplained infertility” should be aware of their chances for future spontaneous conceptions after concluding their treatment, whether successfully or with failure [22].

## 4. Conclusions

Each couple entering ART treatment should be informed of the chances for spontaneous conception in all the above-mentioned scenarios. Nevertheless, for many non-absolute causes of subfertility, spontaneous conception remains possible, but the probability may be very low in complete anatomical or gamete-absence conditions. All the limitations stemming from the local reality, such as cost, state regulations, and local rules, should be mentioned to give the most accurate view of the overall chances of conception. Practitioners should also provide an individualized estimate when possible; explain uncertainty; compare expectant management, IUI, IVF/ICSI, no treatment, adoption, and child-free living; and revisit counseling after failed cycles and after ART live birth. All of these should be done with an emphasis on sharing knowledge and supporting the patients’ decision-making processes. This, in turn, also lends credibility to the institute that does not claim unearned fame. Whether this comprehensive disclosure is made as an aid to the decision to enter or to leave after a failure, or on the more cheerful side, to be aware of chances for unplanned pregnancy after a successful treatment, this data should be put forward. After an ART live birth, counseling should explicitly address the possibility of future natural conception, desired family size, pregnancy spacing, obstetric risk, and contraception [22]. Patients should not be assumed to understand that a prior infertility diagnosis does not necessarily mean permanent infertility. A special note should make clear that a subsequent spontaneous conception should not, by itself, be interpreted as evidence that prior ART was unnecessary, that referral was premature, or that the original diagnosis was incorrect. Rather, it reflects the probabilistic nature of many forms of subfertility.

Although this review uses the couple as the unit of analysis, counseling should recognize that ART often places a substantial physical burden on the female partner, even when the primary factor is male. Male factor infertility is also heterogeneous and may be evaluable or treatable. Therefore, the term ‘male factor’ should not be interpreted as a single uniform prognosis for spontaneous conception.

## Figures and Tables

**Table 1:** Spontaneous conception in ART patients: Summary by clinical windows.

Clinical Window	Study	Design	Denominator	Outcome Definition	Follow-Up Duration	Spontaneous Conception or Live-Birth Estimate
Before ART	Haney et al. (1987) [3]	Enrolled cohort	245 couples	Spontaneous conception	Short	4 conceptions
	Matorras et al. (1996) [4]	Follow-up	86/33/166 couples	Spontaneous pregnancy	2 years	7.6% (0% in azoospermia)
	Eijkemans et al. (2008) [5]	Prospective cohort	5962 couples	Treatment-free pregnancy	1 year	9% cumulative rate
	van Dongen et al. (2010) [6]	Waiting list	674 couples	Dropout/conception	Waiting period	37% of dropouts
Between ART Cycles	Roh et al. (1987) [7]	Comparative	274 women	Indep. conception	2 years	6.6% (83.3% < 6 mo)
	Harrison et al. (1995) [8]	Treatment	662 couples	Spontaneous pregnancy	Not specified	78 couples (16.5% vs. 5% mis)
	Almagor et al. (2001) [9]	Cohort	85 couples	Clinical pregnancy	Waiting period	6.5% (7 pregnancies)
	Donckers et al. (2011) [10]	Consecutive	888 couples	Spontaneous pregnancy	Long-term	24.7% (220 couples)
	Patil (2022) [11]	Retrospective	329 patients	Spontaneous pregnancy	11 months	9% (31 patients)
After ART Birth	Shimizu et al. (1999) [12]	Documented	299 deliveries	Cum. conception	5 years	18%
	Hennelly et al. (2000) [13]	Survey	530 couples	Spontaneous pregnancy	8 years	20.7% (2.5% ICSI)
	Ludwig et al. (2008) [14]	Mail survey	1614 couples	Spontaneous pregnancy	Not specified	20%
	Pinborg et al. (2009) [15]	Prospective	1338 couples	Spontaneous delivery	5 years	18.2%
	Troude et al. (2012) [16]	Survey	2134 couples	Birth rate	7–9 years	17%
	Wynter et al. (2013) [17]	Questionnaire	297 women	Spontaneous conception	2 years	33%
	Lande et al. (2012) [18]	Retrospective	102 couples	Spontaneous live birth	7 years	21%
	Volgsten & Schmidt (2017) [19]	Follow-up	N/A	Conception/Birth	Up to 5 years	26.1%
	El Mokhallalati (2019) [20]	Retrospective	2133 couples	Live birth	5 years	15%
	Alley et al. (2022) [21]	Retrospective	2915 couples	Live birth	Long-term	22%
	Thwaites et al. (2023) [22]	Meta-analysis	5180 couples	Delivery rate	2–15 years	20%
	Başbuğ et al. (2024) [23]	Comparative	48 women	Spontaneous/ICSI	Retrospective	GDM high (spont)
After discontinued	Ben-Rafael (1981) [24]	Clinical	149 patients	Spontaneous conception	Short	0% (tubal); 17.5% (patent)
	Haney et al. (1987) [3]	Enrolled	245 couples	Spontaneous conception	Short	12 conceptions
	Osmanagaoglu (1999) [25]	Follow-up	149 couples	Spontaneous conception	Up to 5 years	1.5% (23 couples)
	Filetto & Makuch (2005) [26]	Interview	76 couples	Spontaneous conception	3–8 years	10.5% (8 women)
	Osmanagaoglu (2002) [27]	Follow-up	200 couples	Delivery	5 years	11.5% (23 deliveries)
	Cahill et al. (2005) [28]	Survey	116 couples	Natural conception	Median 7 mo	18%
	De La Roche. (2009) [29]	Survey	216 reached	Spontaneous conception	~5 years	19% (41 conceived)
	Brandes et al. (2011) [1]	Cohort	104 couples	Spontaneous conception	6–8 years	15.4% (doc)/42.3% (self)
	Troude et al. (2012) [16]	Questionnaire	814 couples	Birth rate	Not specified	24%
	Troude et al. (2016) [30]	Multicenter	6507 couples	Spontaneous conception	8 years	12%
	Marcus et al. (2016) [31]	Web survey	403 couples	Cumulative LB	6 years	29%
	Volkstein & Schmidt [19]	Fragmented	Small group	Birth rate	Not specified	32.9%
	Alley et al. (2022) [21]	Recall	431 couples	Spontaneous conception	<2 months	7.9% (34 conceived)
	Pacchiarotti (2020) [33]	Lockdown	50 couples	Spontaneous conception	2 months	14% (7 conceived)

## Data Availability

Data will be made available to the editors of the journal pre and/or post-publication for review or query upon request.
